# Pomalidomide enhanced gemcitabine and nab-paclitaxel on pancreatic cancer both *in vitro* and *in vivo*

**DOI:** 10.18632/oncotarget.24608

**Published:** 2018-03-02

**Authors:** Nobuhiro Saito, Yoshihiro Shirai, Tadashi Uwagawa, Takashi Horiuchi, Hiroshi Sugano, Koichiro Haruki, Hiroaki Shiba, Toya Ohashi, Katsuhiko Yanaga

**Affiliations:** ^1^ Department of Surgery, The Jikei University School of Medicine, Tokyo, Japan; ^2^ Division of Gene Therapy, Research Center for Medical Science, The Jikei University School of Medicine, Tokyo, Japan; ^3^ Division of Clinical Oncology and Hematology, Department of Internal Medicine, The Jikei University School of Medicine, Tokyo, Japan

**Keywords:** IMiDs, pancreatic cancer, gemcitabine/nab-paclitaxel, NF-κB, p53

## Abstract

**Background:**

Chemotherapy with gemcitabine and nab-paclitaxel (gemcitabine/nab-paclitaxel) is recommended for unresectable pancreatic cancer. However, the therapeutic efficacy is attenuated by the antitumor agent-induced activation of nuclear factor-κB (NF-κB). Thalidomide inhibits NF-κB activation, therefore, we hypothesized that pomalidomide, a third-generation IMiD, would also inhibit NF-κB activation and enhance the antitumor effects of gemcitabine/nab-paclitaxel.

**Methods:**

*In vitro*, we assessed NF-κB activity and apoptosis in response to pomalidomide alone, gemcitabine/nab-paclitaxel, or combination of pomalidomide and gemcitabine/nab-paclitaxel in human pancreatic cancer cell lines (PANC-1 and MIA PaCa-2). *In vivo*, we established orthotopic model and the animals were treated with oral pomalidomide and injection of gemcitabine/nab-paclitaxel.

**Results:**

In pomalidomide and gemcitabine/nab-paclitaxel group, gemcitabine/nab-paclitaxel-induced NF-κB activation was inhibited and apoptosis was enhanced in comparison with those in the other groups both *in vitro* and *in vivo*. Especially, this study revealed for the first time that pomalidomide enhances p53 on pancreatic cancer cells. The tumor growth in the pomalidomide and gemcitabine/nab-paclitaxel group was significantly slower than that in the gemcitabine/nab-paclitaxel group. Moreover, pomalidomide induced G0/G1 cell cycle arrest and suppressed angiogenesis.

**Conclusions:**

Pomalidomide enhanced the antitumor effect of gemcitabine/nab-paclitaxel by inhibition of NF-κB activation. This combination regimen would be a novel strategy for treating pancreatic cancer.

## INTRODUCTION

Pancreatic cancer is one of the most lethal and aggressive of all malignancies and the fourth leading cause of death in the developed countries [1]. Currently, chemotherapy with gemcitabine and nab-paclitaxel (GN) is recommended for locally advanced unresectable and metastatic pancreatic cancer. However, the median overall survival remains unsatisfactory with 8.5 months [2, 3].

Immunomodulatory drugs (IMiDs) have been considered as effective agents for treatment of colorectal [4, 5], lung [6], and prostate cancers [7]. The detailed mechanism by which IMiDs induce apoptosis in solid tumors remains unclear. Pomalidomide (PMD) is a third-generation IMiD derived from thalidomide and has antiproliferative effects on hematological malignancies [8]. Similar to thalidomide, PMD has direct antiproliferative, pro-apoptotic, and antiangiogenetic effects on multiple myeloma cells, as well as modulatory effects on the immune system [8–10].

IMiDs modulate serum levels of inflammatory cytokines and growth factors, such as IL-6, IL-12, IL-8 and granulocyte-macrophage colony-stimulation factor (GM-CSF), which contribute to cancer progression and antitumor immunity [11]. IMiDs enhance the cancer suppressing effect of natural killer (NK) and CD8+ cells by increasing IL-2 levels [12] and by suppression of T regulatory (T-reg) cell [13]. Moreover, IMiDs, including PMD, have been reported to have antitumor effect through induction of apoptosis [8], cell cycle arrest [14] and suppression of angiogenesis [15] in hematological malignancies. PMD has an immunosuppressive effect 15,000 to 50,000 times more potent than thalidomide and 10-fold more potent than lenalidomide on inhibition of tumor necrosis factor (TNF-α) [8, 16]. Therefore, PMD is expected to have more potent antitumor activity as compared to thalidomide or lenalidomide in digestive cancer. In the past clinical trial, the combination of gemcitabine with lenalidomide showed no significant difference as compared to gemcitabine alone [17] on pancreatic cancer. However, considering the strength of the effects of pomalidomide, we believe that GNP therapy is expected to have a stronger effect than the combination of gemcitabine with lenalidomide.

Furthermore, PMD directly induces caspase-8-dependent apoptosis and inhibits nuclear factor-kappaB (NF-κB) transcriptional activity [18, 19]. NF-κB positively acts on the proliferation of digestive cancers through expression of VEGF [20, 21], inhibition of apoptosis proteins (IAPs) [22–24], and induction of chemoresistance [25–27]. Therefore, suppression of NF-κB activation is important to improve chemo-susceptibility. Also, PMD induces cell cycle arrest at the G0/G1 phase in multiple myeloma cells [14]. However, these effects and mechanisms remain unverified in solid tumors.

The purpose of this study is to investigate the antitumor effect and to clarify the mechanisms of PMD in pancreatic cancer.

## RESULTS

### PMD suppressed the activation of nuclear NF-κB *in vitro*

**Figure 1:**
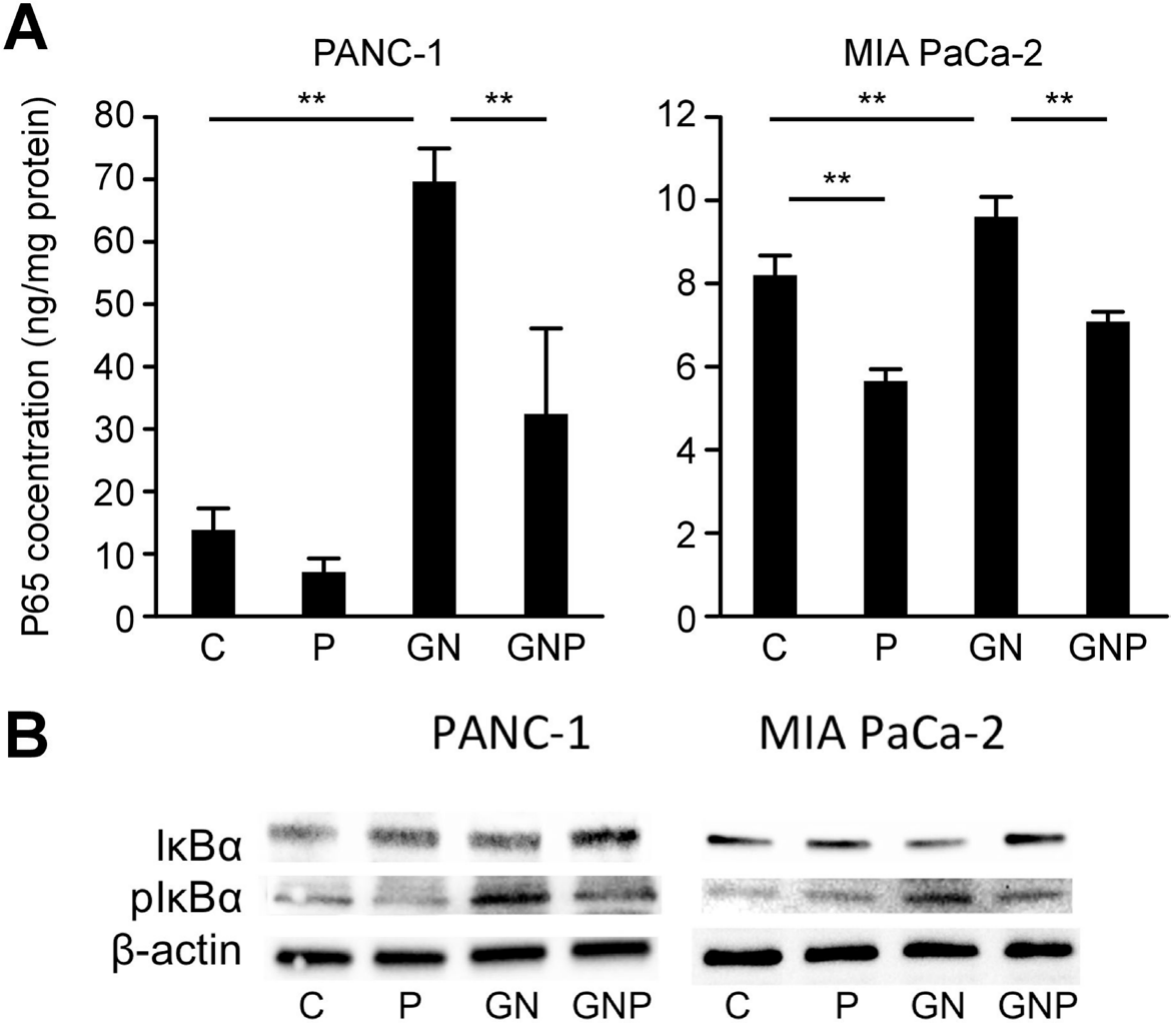
(**A**) The activation of NF-κB was measured using ELISA at 2 hours after each treatment. The concentrations of NF-κB p65 in the nuclear extracts of PANC-1 and MIA PaCa-2 in GN group were significantly greater than those in C group (PANC-1, *p* < 0.01; MIA PaCa-2, *p* < 0.01), whereas those in GNP group were significantly lower than those in GN group (PANC-1, *p* < 0.01; MIA PaCa-2, *p* < 0.01). (**B**) Similar to nuclear NF-κB concentrations, IκBα levels of PANC-1 and MIA PaCa-2 in P group were greater than those in C group, whereas phosphorylated IκBα levels of PANC-1 and MIA PaCa-2 in GNP group were lower than those in GN group at 2 hours after treatment.

To evaluate the activation of NF-κB in pancreatic cancer cell lines, the concentration of p65 in the nuclear extracts were measured using ELISA, and IκBα levels were evaluated using Western blot analysis. The concentrations of NF-κB p65 in the nuclear extracts of PANC-1 and MIA PaCa-2 in the GN group (GN group means cells treated with GN) were significantly greater than those in the C group (*p* < 0.01) (C group means cells treated with vehicle), whereas those in the GNP group (GNP group means cells treated with GNP) were significantly lower than those in the GN group (*p* < 0.01). In MIA PaCa-2 cells, the concentrations of NF-κB p65 in the nuclear extracts in the P group were significantly lower than that in the C group (*p* < 0.01, Figure 1A) (P group means cells treated with PMD alone). Similar to nuclear NF-κB concentrations, IκBα levels of PANC-1 and MIA PaCa-2 in the P group were greater than those in the C group, whereas phosphorylated IκBα levels of PANC-1 and MIA PaCa-2 in the GNP group were lower than those in the GN group (Figure 1B).

### PMD enhanced antitumor effects and induced apoptosis

**Figure 2:**
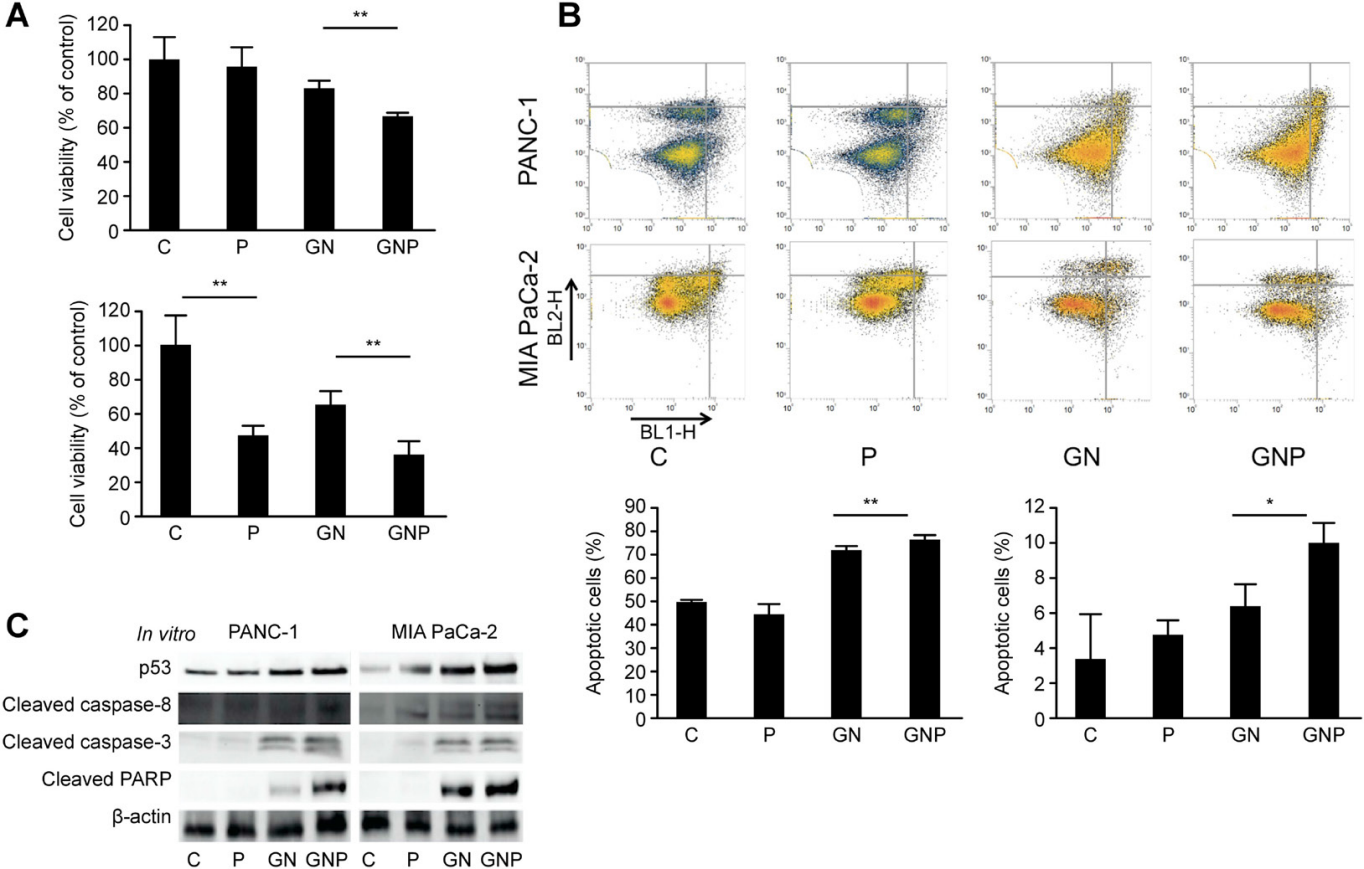
(**A**) Cell viabilities of PANC-1 and MIA PaCa-2 cells were shown as the percent of control at 72 hours (PANC-1) and 48 hours (MIA PaCa-2) after treatment. In the cell proliferation assay, the viability in GNP group of PANC-1 and MIA PaCa-2 was significantly lower than that in GN group (PANC-1, *p* < 0.01; MIA PaCa-2, *p* < 0.01). (**B**) The proportion of early and late apoptotic cells was evaluated by Annexin/FITC analysis at 72 hours (PANC-1) and 48 hours (MIA PaCa-2) after treatment. The apoptotic cells in GNP group of PANC-1 and MIA PaCa-2 were significantly greater than those in GN group (PANC-1, *p* < 0.01; MIA PaCa-2, *p* < 0.05). (**C**) Western blot analysis demonstrated the expression of proteins related to apoptosis. After 2 hours treatment with PMD, gemcitabine and nab-paclitaxel were administered. PANC-1 and MIA PaCa-2 cells were harvested at 72 hours and 48 hours after treatment, respectively. The levels of p53, cleaved caspase-8 and -3 as well as cleaved PARP in GNP group were greater than those in GN group.

To evaluate the antitumor effect of PMD in pancreatic cancer, we examined cell viability and apoptotic cells. In the cell proliferation assay, the viability of MIA PaCa-2 cells was significantly decreased by PMD monotherapy (46.9 ± 6.2% vs. 100 ± 17.0%, *p* < 0.01) and the viabilities in the GNP group of PANC-1 and MIA PaCa-2 were significantly lower than those in the GN group (PANC-1, 67.0 ± 1.6% vs. 83.2 ± 4.3%, *p* < 0.01; MIA PaCa-2: 35.5 ± 8.3% vs. 64.8 ± 7.9%, *p* < 0.01, Figure 2A). In the apoptosis analysis, the apoptotic cells in the GNP group of PANC-1 and MIA PaCa-2 were significantly greater than those in the GN group (PANC-1, 77.1 ± 0.9% vs. 72.0 ± 1.3%, *p* < 0.01; MIA PaCa-2, 10.0 ± 1.1% vs. 6.44 ± 1.2%, *p* < 0.05, respectively, Figure 2B). To investigate more details of the molecular mechanism by which PMD induced apoptosis, the apoptotic signals were evaluated using Western blot analysis. The levels of p53, cleaved caspase-8 and -3 as well as cleaved PARP in the GNP group were greater than those in the GN group (Figure 2C).

### *In vivo* analysis

**Figure 3:**
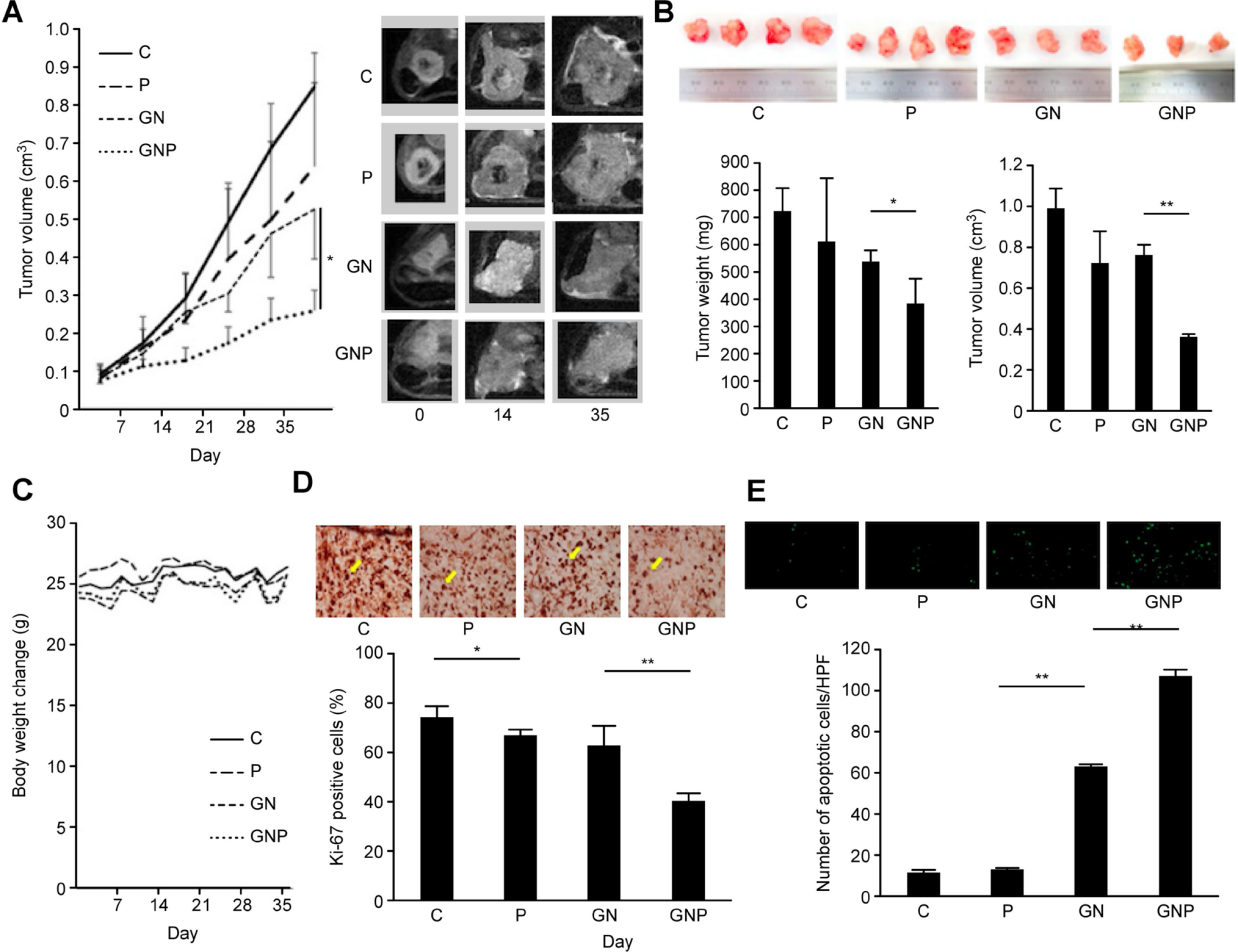
(**A**) T2-weighted MRI detected tumors in the tail of the pancreas on the left side of the body. MRI revealed that tumor growth in GNP group was slower than that in GN group (*p* < 0.05). (**B**) At five weeks after treatment, both tumor weight and volume of excised tumor in GNP group were significantly lower than those in GN group (weight, *p* < 0.05; volume, *p* < 0.01) (*n* = 4 each). (**C**) Body weight changes during the treatment. There was no difference in the change in body weight among the experimental groups. (**D**) In GNP group, the number of Ki-67-positive cells in the excised tumor was significantly lower than that in GN group (*p* < 0.01). The number of Ki-67-positive cells in P group was significantly lower than that in C group (*p* < 0.05) (×400). (**E**) The TUNEL assay of the excised tumor showed that the number of apoptotic cells in GNP group was significantly greater than that in GN group (*p* < 0.01) (×400).

The tumor growth in the GNP group was significantly slower than that in the GN group (Figure 3A). Both tumor weights and volume of the excised tumor in the GNP group were significantly lower than those in GN group (weight, 385.3 ± 88.2 mg vs. 538.6 ± 36.2 mg, *p* < 0.05; volume, 0.36 ± 0.02 cm^3^ vs. 0.76 ± 0.04 cm^3^, *p* < 0.01, respectively, *n* = 4 for each, Figure 3B). In contrast, there was no difference in the change in body weight among the treatment groups (Figure 3C). In the GNP group, the number of Ki-67-positive cells in the excised tumor was significantly lower than that in the GN group (40.7 ± 3.2 vs. 63.3 ± 7.7 %, *p* < 0.01). Additionally, the number of Ki-67-positive cells in the P group was significantly lower than that in the C group (67.1 ± 2.4% vs. 74.8 ± 3.8%, *p* < 0.05, Figure 3D). The TUNEL assay of the excised tumor showed that the number of apoptotic cells in the GNP group was significantly greater than that in the GN group (107 ± 2.6 vs. 63.0 ± 1.0, *p* < 0.01, Figure 3E).

**Figure 4:**
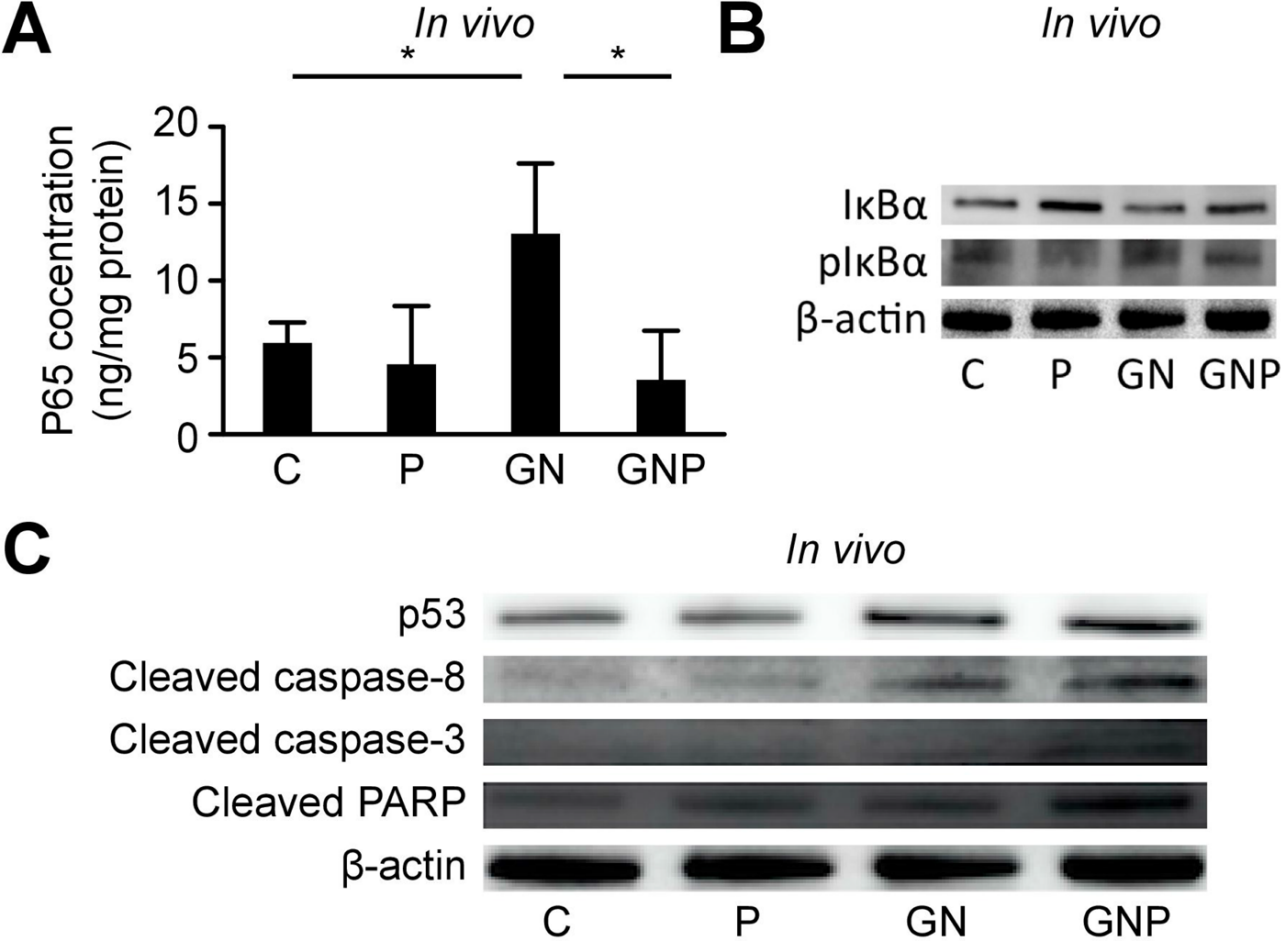
(**A**) The concentrations of NF-κB p65 in the excised tumor in GN group were significantly greater than those in C group (*p* < 0.05), whereas those in GNP group were significantly lower than those in GN group (*p* < 0.05). (**B**) Similar to nuclear NF-κB concentrations, IκBα levels in P group were greater than those in C group, whereas phosphorylated IκBα levels in GNP group were lower than those in GN group, as observed in the *in vitro* experiments. (**C**) Similar to the *in vitro* experiments, the level of p53, cleaved caspase-8 and 3 as well as cleaved PARP in GNP group were greater than those in the other groups.

NF-κB activation in the excised tumor was assessed using ELISA, and IκBα levels were evaluated using Western blot analysis. The concentrations of NF-κB p65 in the excised tumor in the GN group were significantly greater than those in the C group (*p* < 0.05), whereas those in the GNP group were significantly lower than those in the GN group (*p* < 0.05, Figure 4A). Similar to nuclear NF-κB concentrations, IκBα levels in the P group were greater than those in the C group, whereas phosphorylated IκBα levels in the GNP group were lower than those in the GN group, as observed in the *in vitro* experiments (Figure 4B). Similar to the *in vitro* experiments, the level of p53, cleaved caspase-8 and 3 as well as cleaved PARP in the GNP group were greater than those in the other groups (Figure 4C).

### G0/G1 cell cycle arrest

**Figure 5:**
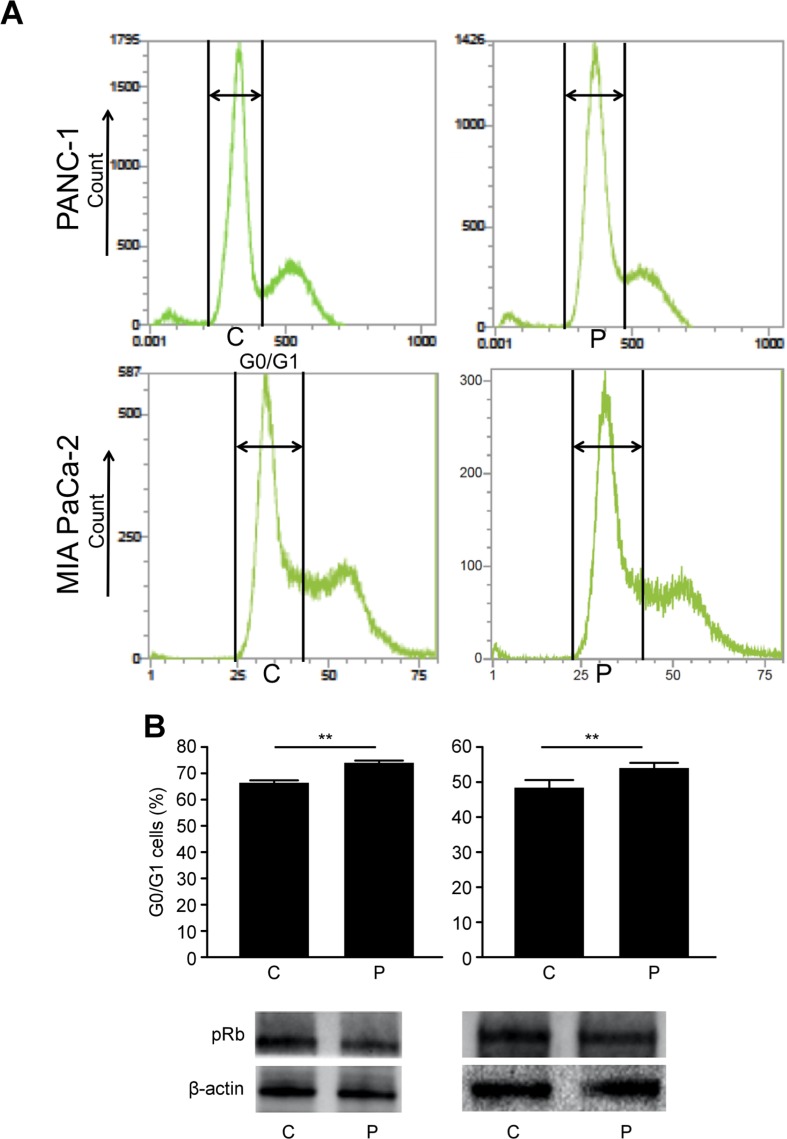
(**A**) The proportion of cells in the G0/G1 phase in P group was greater than that in C group (PANC-1, *p* < 0.01; MIA PaCa-2, *p* < 0.01). (**B**) The level of phosphorylated Rb (Ser 780) protein in P group was lower than that in C group for both PANC-1 and MIA PaCa-2 cells.

To evaluate the effect of PMD on cell cycle progression in pancreatic cancer cells, we performed cell cycle analysis using flow cytometry. The proportion of cells in the G0/G1 phase in the P group was greater than that in the C group (PANC-1, 74.0 ± 1.0% vs. 66.3 ± 1.0%, *p* < 0.01; MIA PaCa-2, 54.2 ± 1.4% vs. 48.6 ± 2.2%, *p* < 0.01, Figure 5A). Additionally, the expression levels of phosphorylated Rb, which plays an important role in cell cycle progression [28, 29] was examined. The level of phosphorylated Rb (Ser 780) protein in the P group was lower than that in the C group for PANC-1 and MIA PaCa-2 (Figure 5B). In this experiment, it was not appropriate to correctly evaluate the influence of PMD on GN with flow cytometry because GN also influence on cell cycle. Therefore, we simply examined the effect of PMD on pancreatic cancer cells.

### PMD inhibited angiogenesis in pancreatic cancer cells

**Figure 6:**
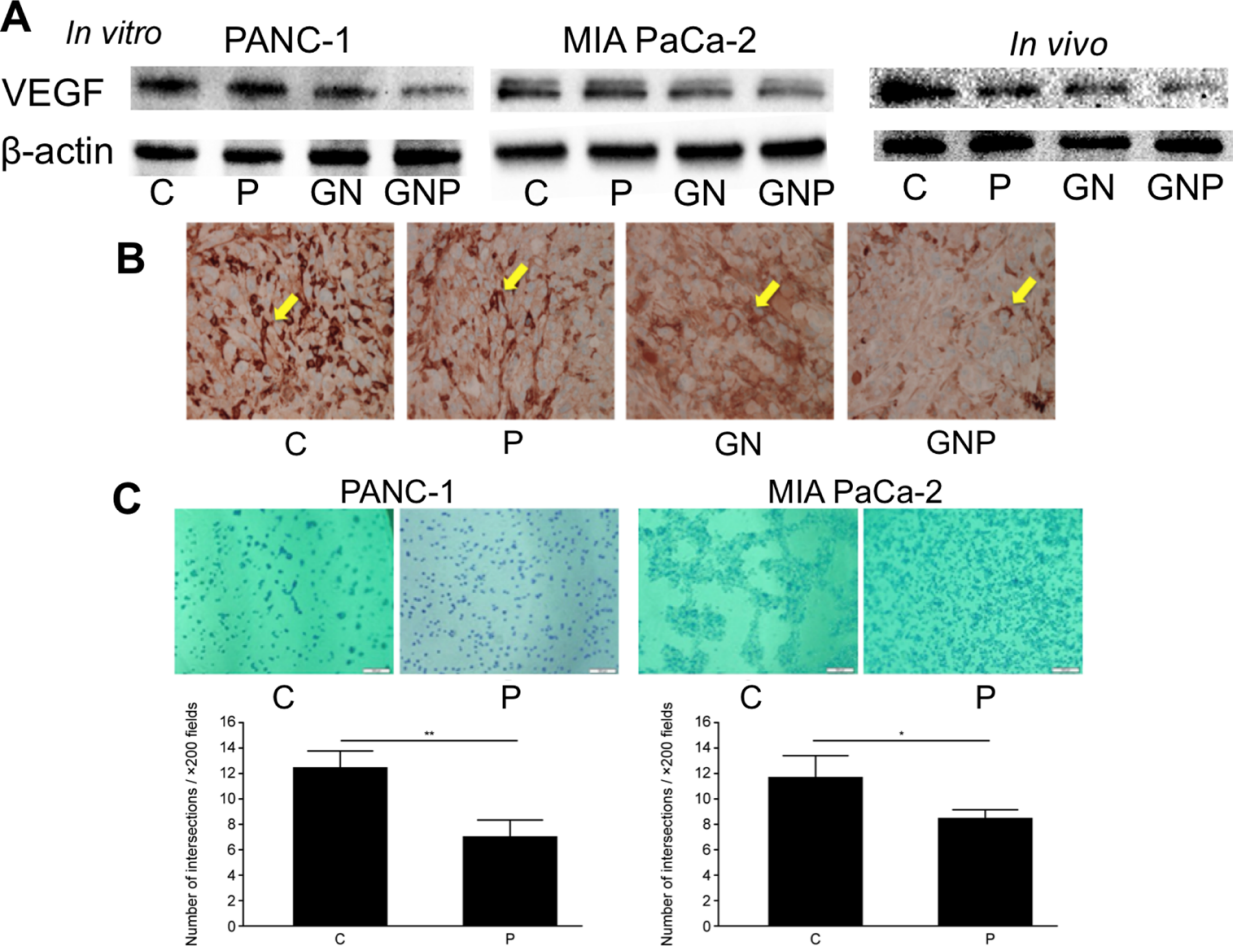
(**A**) The levels of VEGF in GNP group were lower than those in GN group, both *in vitro* and *in vivo.* (**B**) Immunohistochemical staining of excised tumors showed that the number of VEGF-positive cells in GNP group was fewer than those in GN group (×400). (**C**) The *in vitro* angiogenesis assay revealed the number of connected cells in P group was significantly fewer than that in C group (PANC-1, *p* < 0.01; MIA PaCa-2, *p* < 0.05) (×200).

To investigate detailed molecular mechanism of antitumor effect by PMD, the expression of VEGF, which plays an important role in angiogenesis and is a target molecule of NF-κB was measured. The levels of VEGF protein in the GNP group were lower than those in the GN group, both *in vitro* and *in vivo* (Figure 6A). Immunohistochemical staining of the excised tumors showed that the number of VEGF-positive cells in the GNP group was fewer than that in the GN group (Figure 6B). The angiogenesis assay was performed to evaluate the effect of PMD on endothelial cell tube formation in PANC-1 and MIA PaCa-2. The number of connected cells in the P group was significantly fewer than that in the C group (PANC-1, 7.1 ± 1.3 vs. 12.5 ± 1.3, *p* < 0.01; MIA PaCa-2, 8.4 ± 0.6 vs. 11.8 ± 1.6, *p* < 0.05, respectively, Figure 6C). This experiment was not suitable for comparing GN and GNP group because all the cells in both GN group and GNP group were separated with each other. Therefore, we compared C group and P group.

## DISCUSSION

Recently, combination chemotherapy with gemcitabine and nab-paclitaxel has been demonstrated to be effective for unresectable pancreatic cancer [30–32]. However, antitumor drug-induced NF-κB activation is considered to reduce chemosensitivity thorough various inflammation-related gene transcription [33]. Therefore, suppression of agent-induced NF-κB activation could be a new therapeutic approach to improve chemosensitivity [22, 23, 25, 32, 34–36].

In the current study, GN decreased IκBα and enhanced NF-κB activation, whereas PMD suppressed phosphorylation of IκBα and inhibited the NF-κB activation. Moreover, PMD had dual apoptotic effect of enhancing p53 and cleaved caspase-8-dependent pathway on pancreatic cancer. To the best of our knowledge, we firstly demonstrated enhancement of p53, which directly activates the transcription of apoptotic genes [37, 38] by PMD on pancreatic cancer.

PMD is also known to induce G0/G1 phase cell cycle arrest [14]. The pRb regulates the local chromatin structure by recruiting histone deacetylase 1 (HDAC1) to modulate the balance of histone acetylation levels. The pRb blocks cell cycle progression by repressing E2F-target gene transcription through the recruitment of transcriptional corepressors and chromatin remodeling protein factors, such as HDACs, Sin3, CtBP and SWI/SNF, at promoter regions [28]. Hypophosphorylated pRb binds to DNA-bound E2F transcriptional regulators and suppresses their target gene expression through the recruitment of HDACs, co-repressors and chromatin remodeling enzymes. When it is in a hyperphosphorylated state, the complex is dissociated, and the cell cycle progresses [39, 40]. In the current study, we firstly demonstrated that PMD inhibited the Ser^780^ phosphorylation of pRb and caused G0/G1 cell cycle arrest. These result suggested that PMD contributed to G0/G1 cell cycle arrest via the pRb pathway in pancreatic cancer cells.

The importance of angiogenesis in cancer progression has been demonstrated in various cancers [41]. VEGF is downstream of NF-κB signal transduction and is known to play a key role in neovascularization [42, 43]. In the current study, we have shown that the level of VEGF was decreased and neovascularization was suppressed by PMD. Regarding solid tumor, previous report has also described that IMiDs inhibit angiogenesis in gastric cancer [44].

PMD has a manageable toxicity profile in patients who relapsed after multiple types of therapy for myeloma [45]. The most common grade 3 or 4 hematologic side effect is neutropenia, while non-hematologic grade 3 or 4 adverse effects are fatigue and pneumonia [8]. Grades 3 and 4 adverse events were reported in more than 5% of patients in the phase II clinical trials for PMD/dexamethasone for multiple myeloma [8]. Our *in vivo* study showed no significant body weight differences or treatment-related deaths. This indicated that current regimen does not have nutrition-related disadvantages.Nevertheless, another *in vivo* study using PMD shown thrombocytosis in combination with gemcitabine and S-1, therefore we must keep in mind checking platelet count in clinical use.

In summary, PMD enhanced the antitumor effects of GN by inhibiting NF-κB activation. PMD induced caspase-8-mediated apoptosis, enhancement of p53, cell cycle arrest, and antiangiogenesis. Therefore, PMD with GN therapy may be a novel treatment with strong antitumor effect on pancreatic cancer.

## MATERIALS AND METHODS

### Cell culture

The human pancreatic cancer cell lines PANC-1 and MIA PaCa-2 were purchased from the American Type Culture Collection (ATCC, Rockville, MD). PANC-1 and MIA PaCa-2 were guaranteed by ATCC, and maintained in Dulbecco’s modified Eagle’s Medium (D-MEM) (Wako Pure Chemical Industries, Ltd., Osaka, Japan) containing 10% fetal bovine serum (Gibco, Grand Island, NY) and 1% penicillin/streptomycin (Gibco/Brl). The cells were cultured at 37° C with 5% CO_2_.

### Reagents

PMD was purchased from Tokyo Chemical Industry (Tokyo, Japan) and was dissolved in 1% dimethyl sulfoxide (DMSO) and stored at −80° C until use. Gemcitabine was purchased from Eli Lilly Japan (Kobe, Japan). Nab-paclitaxel was purchased from Taiho Pharmaceutical Co. Ltd (Tokyo, Japan).

### Antibodies

Monoclonal antibodies to inhibitor of kappaBα (IκBα), cleaved caspase-3 and -8, and cleaved poly-ADP-ribose polymerase (PARP) were purchased from Cell Signaling Technology (Beverly, MA). Anti-b-actin antibody was purchased from Sigma-Aldrich (St. Louis, MO). Monoclonal antibodies against p53 (DO-1) and VEGF (A-20) were purchased from Santa Cruz Biotechnology, Inc (Santa Cruz, CA). Anti-Rb (phospho-Ser 780) antibody was purchased from Abcam (Cambridge, UK). Monoclonal anti-Ki-67 antibody (MIB-1) was purchased from Dako (Glostrup, Denmark).

### Animals

Five-week-old male nude mice (BALBc nu/nu) were purchased from CLEA Japan, Inc, (Tokyo, Japan) and housed under specific pathogen-free conditions in a biological cabinet at the Laboratory Animal Facility of the Jikei University School of Medicine. The protocol for the animal experiments was approved by the Institutional Animal Care and Use Committee of Jikei University (no. 2015–002) and fulfilled the Guidelines for the Proper Conduct of Animal Experiments of the Science Council of Japan (2006). All the guidelines above are based on the Declaration of Helsinki.

### *In vitro* experimental protocol

PANC-1 and MIA PaCa-2 cells were treated in 4 conditions: pomalidomide (100 μM; P group), gemcitabine (1000 nM and 494 nM, respectively) and nab-paclitaxel (500 nM and 683 nM, respectively) (GN group), gemcitabine and nab-paclitaxel with pomalidomide (GNP group), or vehicle only (1% DMSO; C group) for the appropriate time in each analysis. The concentrations of gemcitabine and nab-paclitaxel were determined according to the previous studies [30, 32]. The appropriate doses were determined with reference to the IC50 and different concentrations of experiments we performed. In the GNP group, the cells were treated with PMD for 2 hours before the administration of gemcitabine and nab-paclitaxel.

### *In vivo* experimental animal model and protocol

The orthotopic pancreatic cancer mice model was used as previously described [32, 46]. The mice were continuously anesthetized with 2% isoflurane (Pfizer Japan Inc., Tokyo, Japan), and a 5-mm incision was made in the left flank on the splenic silhouette. The distal pancreas was gently delivered from the peritoneal cavity with spleen thorough the incision. 5.0 × 10^6^ PANC-1 cells suspended in 40 µl of phosphate buffered saline (PBS) were injected into the tail of the pancreas. After injection, the pancreas was placed in the abdomen and the abdominal wound site was closed in two layers. At 4 weeks after the injection, the animals were randomly divided into four treatment groups, which were the same as the *in vitro* experimental groups (*n* = 4). Pomalidomide (0.5 mg/kg) was administered orally five times a week, and gemcitabine (50 mg/kg) and nab-paclitaxel (0.5 mg/kg) were injected intraperitoneally once a week. The doses of antitumor agents were determined according to the previous studies [32, 47]. In the C group, vehicle of pomalidomide (PBS) was administered orally five times a week, which vehicles of gemcitabine and nab-paclitaxel (distilled water and isotonic sodium, respectively) were injected intraperitoneally once a week. The tumor volume was evaluated sequentially once a week by magnetic resonance imaging (MRI). At five weeks after treatment, the animals were sacrificed and orthotopic pancreatic tumors were excised for the assessment.

### MRI *in vivo* protocol

The animals were imaged in the prone position inside a clinical 9.4 T MRI System (BioSpin 94/20 USR, Bruker, Ettingen, Germany) using a coronal multi-slice T2-weighted protocol (TurboRARE-T2) (Paravision 5.1; PV5.1, Bruker).

### Quantitative analysis of NF-κB activity

NF-κB is typically a heterodimer consisting of the p65 and p50 proteins, and it normally resides in the cytoplasm as an inactivate form as a complex with an IκBα. NF-κB activation signals lead to IκBα phosphorylation, and NF-κB is released from IκBα, and then translocated into the nucleus. Nuclear extracts from both *in vitro* and *in vivo* experiments were prepared using a nuclear extract kit (Active Motif, Carlsbad, CA) according to the manufacturer’s instructions. The nuclear extracts were assayed using an enzyme-linked immunosorbent assay (ELISA) kit (Trans-AM NF-κB p65 Activation Assay; Active Motif) to detect and quantify the NF-κB p65 activity according to the manufacturer’s instructions. The levels of NF-κB p65 were measured at 2 hours after treatment.

### Western blot analysis

Whole protein extracts from both *in vitro* and *in vivo* experiments were prepared according to the protocol described previously [48]. The lysate protein was extracted by 2% sodium dodecyl sulfate, electrophoresed on 4% to 20% acrylamide gradient gels in Tris-glycine buffer, and transferred onto a nitrocellulose membrane. After incubating the blots in each primary antibody (1:1000 dilution) overnight, the membranes were then incubated with secondary antibody (1:10,000 dilution, Histofine; Nichirei, Tokyo, Japan). A luminol enhancer and peroxide solution, Clarity Max (Bio-Rad Laboratories, Inc., Hercules, CA), was used to detect the protein bands. Protein bands were detected using a Chemi Doc XRS+ system and evaluated using Image Lab software (Bio-Rad).

### Cell proliferation assay

The cells were seeded into 96-well plates (5 × 10^3^ cells/well) and treated for 72 hours (PANC-1) or 48 hours (MIA PaCa-2). The cell proliferation was measured using a CellTiter-Blue Cell Viability Assay Kit (Promega, Madison, WI) according to the manufacturer’s instructions. The stained cells were analyzed with a PerkinElmer EnSpire plate reader (PerkinElmer Inc., Waltham, MA).

### Assessment of apoptosis by flow cytometry

The cells (1 × 10^6^ cells) were seeded and treated with each regimen for 72 hours (PANC-1) or 48 hours (MIA PaCa-2). These cells were washed with 1× binding buffer and incubated with 10 µl of Annexin V/FITC (Annexin V/FITC Kit; Miltenyi Biotec GmbH, Cologne, Germany) for 15 minutes in the dark at room temperature. The stained cells were analyzed with an Attune NxT Flow Cytometer (Thermo Fisher Scientific, Waltham, MA).

### Cell cycle analysis

The cells were harvested after treatment for 72 hours (PANC-1) or 48 hours (MIA PaCa-2) and were fixed in 70% ethanol for 30 minutes at −20° C. After centrifugation, the cell pellets were washed with PBS and incubated at room temperature for 1 minute, followed by incubation with RNase (1 µg/ml) for 4 minutes. Finally, these cells were incubated with propidium iodide (1 mg/ml) (Sigma-Aldrich, St Louis, MO) for 30 minutes. DNA content was determined with an Attune NxT Flow Cytometer (Thermo Fisher Scientific).

### *In vitro* angiogenesis assay

To evaluate the antiangiogenic effect of PMD in pancreatic cancer, an *in vitro* angiogenesis assay kit (Trevigen Inc., Gaithersburg, MD) was used. Pancreatic cell lines were seeded into 96-well plates (5 × 10^3^ cells/well) on top of an aliquot of 50 µl of basement membrane. These cells were incubated with PMD for 24 hours and fixed with 3.7% methanol. These cells were continuously stained with cell staining solution according to the manufacturer’s instructions. This assay evaluates the ability of the cells to connect. The number of intersections was counted in three random fields per well for three wells. This experiment was conducted with reference to [49].

### Immunohistochemical staining

Paraffin sections of tumor tissues were stained immunohistochemically using anti-Ki-67 and VEGF antibodies as the primary antibodies and the DAKO Envision Kit/HRP as a secondary antibody (DAKO, Carpenteria, CA). A TdT-mediated dUTP nick end labeling (TUNEL) assay was performed using an *In Situ* Cell Death Detection Kit with fluorescein (Roche Diagnostics, Basel, Switzerland) to evaluate the induction of apoptosis. These measurements were performed according to the manufacturer’s instructions. The number of Ki-67-positive cells and TUNEL-positive cells were counted in three random fields (×400 and ×200, respectively) in three tumors.

### Statistical analysis

Data are expressed as the mean ± SD. Non-paired t-test (2-tailed) and repeated measurements analysis (ANOVA) of variance were used for statistical analysis with SPSS 23.0 (IBM Japan, Tokyo, Japan). All *p* values were considered statistically significant when the associated probability was < 0.05.

## Abbreviations

PMD: pomalidomide
GN: gemcitabine and nab-paclitaxel therapy
GNP: the combination chemotherapy of pomalidomide with GN
C group: treated with only vehicle
P group: treated with PMD alone
GN group: treated with GN
GNP group: treated with GNP
NF-κB: nuclear factor-κB
IκBα: inhibitor of kB
pIκBα: phosphorylated IκBα
PARP: poly-ADP-ribose polymerase
ELISA: enzyme-linked immunosorbent assay
TUNEL assay: A TdT-mediated dUTP nick end labeling assay

## References

[1] Siegel RL, Miller KD, Jemal A (2017). Cancer Statistics, 2017. CA Cancer J Clin.

[2] Von Hoff DD, Ramanathan RK, Borad MJ, Laheru DA, Smith LS, Wood TE, Korn RL, Desai N, Trieu V, Iglesias JL, Zhang H, Soon-Shiong P, Shi T (2011). Gemcitabine plus nab-paclitaxel is an active regimen in patients with advanced pancreatic cancer: a phase I/II trial. J Clin Oncol.

[3] Von Hoff DD, Ervin T, Arena FP, Chiorean EG, Infante J, Moore M, Seay T, Tjulandin SA, Ma WW, Saleh MN, Harris M, Reni M, Dowden S (2013). Increased survival in pancreatic cancer with nab-paclitaxel plus gemcitabine. N Engl J Med.

[4] Bertino EM, McMichael EL, Mo X, Trikha P, Davis M, Paul B, Grever M, Carson WE, Otterson GA (2016). A Phase I Trial to Evaluate Antibody-Dependent Cellular Cytotoxicity of Cetuximab and Lenalidomide in Advanced Colorectal and Head and Neck Cancer. Mol Cancer Ther.

[5] Liu WM, Henry JY, Meyer B, Bartlett JB, Dalgleish AG, Galustian C (2009). Inhibition of metastatic potential in colorectal carcinoma *in vivo* and *in vitro* using immunomodulatory drugs (IMiDs). Br J Cancer.

[6] Ellis PM, Jungnelius U, Zhang J, Fandi A, Beck R, Shepherd FA (2013). A phase I study of pomalidomide (CC-4047) in combination with cisplatin and etoposide in patients with extensive-stage small-cell lung cancer. J Thorac Oncol.

[7] Amato RJ, Glode LM, Podolnick J, Knight R, Crawford D (2011). Phase II Study of Pomalidomide in Patients with Castration-Resistant Prostate Cancer. Cancers (Basel).

[8] Richardson PG, Mark TM, Lacy MQ (2013). Pomalidomide: new immunomodulatory agent with potent antiproliferative effects. Crit Rev Oncol Hematol.

[9] Quach H, Ritchie D, Stewart AK, Neeson P, Harrison S, Smyth MJ, Prince HM (2010). Mechanism of action of immunomodulatory drugs (IMiDS) in multiple myeloma. Leukemia.

[10] Feng N, Chen H, Fu S, Bian Z, Lin X, Yang L, Gao Y, Fang J, Ge Z (2016). HIF-1alpha and HIF-2alpha induced angiogenesis in gastrointestinal vascular malformation and reversed by thalidomide. Sci Rep.

[11] Galustian C, Meyer B, Labarthe MC, Dredge K, Klaschka D, Henry J, Todryk S, Chen R, Muller G, Stirling D, Schafer P, Bartlett JB, Dalgleish AG (2009). The anti-cancer agents lenalidomide and pomalidomide inhibit the proliferation and function of T regulatory cells. Cancer Immunol Immunother.

[12] Fionda C, Abruzzese MP, Zingoni A, Cecere F, Vulpis E, Peruzzi G, Soriani A, Molfetta R, Paolini R, Ricciardi MR, Petrucci MT, Santoni A, Cippitelli M (2015). The IMiDs targets IKZF-1/3 and IRF4 as novel negative regulators of NK cell-activating ligands expression in multiple myeloma. Oncotarget.

[13] Sakamaki I, Kwak LW, Cha SC, Yi Q, Lerman B, Chen J, Surapaneni S, Bateman S, Qin H (2014). Lenalidomide enhances the protective effect of a therapeutic vaccine and reverses immune suppression in mice bearing established lymphomas. Leukemia.

[14] Hernandez-Garcia S, San-Segundo L, Gonzalez-Mendez L, Corchete LA, Misiewicz-Krzeminska I, Martin-Sanchez M, Lopez-Iglesias AA, Algarin EM, Mogollon P, Diaz-Tejedor A, Paino T, Tunquist B, Mateos MV (2017). The kinesin spindle protein inhibitor filanesib enhances the activity of pomalidomide and dexamethasone in multiple myeloma. Haematologica.

[15] Polizzotto MN, Uldrick TS, Wyvill KM, Aleman K, Peer CJ, Bevans M, Sereti I, Maldarelli F, Whitby D, Marshall V, Goncalves PH, Khetani V, Figg WD (2016). Pomalidomide for Symptomatic Kaposi’s Sarcoma in People With and Without HIV Infection: A Phase I/II Study. J Clin Oncol.

[16] Corral LG, Haslett PA, Muller GW, Chen R, Wong LM, Ocampo CJ, Patterson RT, Stirling DI, Kaplan G (1999). Differential cytokine modulation and T cell activation by two distinct classes of thalidomide analogues that are potent inhibitors of TNF-alpha. J Immunol.

[17] Infante JR, Arkenau HT, Bendell JC, Rubin MS, Waterhouse D, Jones GT, Spigel DR, Lane CM, Hainsworth JD, Burris HA (2013). Lenalidomide in combination with gemcitabine as first-line treatment for patients with metastatic carcinoma of the pancreas: a Sarah Cannon Research Institute phase II trial. Cancer Biol Ther.

[18] Das DS, Ray A, Song Y, Richardson P, Trikha M, Chauhan D, Anderson KC (2015). Synergistic anti-myeloma activity of the proteasome inhibitor marizomib and the IMiD immunomodulatory drug pomalidomide. Br J Haematol.

[19] Davies F, Baz R (2010). Lenalidomide mode of action: linking bench and clinical findings. Blood Rev.

[20] Wang F, Yang JL, Yu KK, Xu M, Xu YZ, Chen L, Lu YM, Fang HS, Wang XY, Hu ZQ, Li FF, Kan L, Luo J (2015). Activation of the NF-kappaB pathway as a mechanism of alcohol enhanced progression and metastasis of human hepatocellular carcinoma. Mol Cancer.

[21] Furukawa K, Iida T, Shiba H, Fujiwara Y, Uwagawa T, Shimada Y, Misawa T, Ohashi T, Yanaga K (2010). Anti-tumor effect by inhibition of NF-kappaB activation using nafamostat mesilate for pancreatic cancer in a mouse model. Oncol Rep.

[22] Furukawa K, Uwagawa T, Haruki K, Fujiwara Y, Iida T, Shiba H, Misawa T, Ohashi T, Yanaga K (2013). Nuclear factor kappaB activity correlates with the progression and prognosis of pancreatic cancer in a mouse model. Surg Today.

[23] Gocho T, Uwagawa T, Furukawa K, Haruki K, Fujiwara Y, Iwase R, Misawa T, Ohashi T, Yanaga K (2013). Combination chemotherapy of serine protease inhibitor nafamostat mesilate with oxaliplatin targeting NF-kappaB activation for pancreatic cancer. Cancer Lett.

[24] Kocab AJ, Veloso A, Paulsen MT, Ljungman M, Duckett CS (2015). Effects of physiological and synthetic IAP antagonism on c-IAP-dependent signaling. Oncogene.

[25] Uwagawa T, Chiao PJ, Gocho T, Hirohara S, Misawa T, Yanaga K (2009). Combination chemotherapy of nafamostat mesilate with gemcitabine for pancreatic cancer targeting NF-kappaB activation. Anticancer Res.

[26] Furukawa K, Iida T, Shiba H, Fujiwara Y, Uwagawa T, Shimada Y, Misawa T, Ohashi T, Yanaga K (2010). Anti-tumor effect by inhibition of NF-κB activation using nafamostat mesilate for pancreatic cancer in a mouse model. Oncology Reports.

[27] Lau MC, Ng KY, Wong TL, Tong M, Lee TK, Ming XY, Law S, Lee NP, Cheung AL, Qin YR, Chan KW, Ning W, Guan XY (2017). FSTL1 Promotes Metastasis and Chemoresistance in Esophageal Squamous Cell Carcinoma through NFkappaB-BMP Signaling Cross-talk. Cancer Res.

[28] Uchida C (2016). Roles of pRB in the regulation of nucleosome and chromatin structures. Biomed Res Int.

[29] Singh SK, Banerjee S, Acosta EP, Lillard JW, Singh R (2017). Resveratrol induces cell cycle arrest and apoptosis with docetaxel in prostate cancer cells via a p53/ p21WAF1/CIP1 and p27KIP1 pathway. Oncotarget.

[30] Awasthi N, Zhang C, Schwarz AM, Hinz S, Wang C, Williams NS, Schwarz MA, Schwarz RE (2013). Comparative benefits of Nab-paclitaxel over gemcitabine or polysorbate-based docetaxel in experimental pancreatic cancer. Carcinogenesis.

[31] Frese KK, Neesse A, Cook N, Bapiro TE, Lolkema MP, Jodrell DI, Tuveson DA (2012). nab-Paclitaxel potentiates gemcitabine activity by reducing cytidine deaminase levels in a mouse model of pancreatic cancer. Cancer Discov.

[32] Horiuchi T, Uwagawa T, Shirai Y, Saito N, Iwase R, Haruki K, Shiba H, Ohashi T, Yanaga K (2016). New treatment strategy with nuclear factor-kappaB inhibitor for pancreatic cancer. J Surg Res.

[33] Uwagawa T, Li Z, Chang Z, Xia Q, Peng B, Sclabas GM, Ishiyama S, Hung MC, Evans DB, Abbruzzese JL, Chiao PJ (2007). Mechanisms of synthetic serine protease inhibitor (FUT-175)-mediated cell death. Cancer.

[34] Keifer JA, Guttridge DC, Ashburner BP, Baldwin AS (2001). Inhibition of NF-kappa B activity by thalidomide through suppression of IkappaB kinase activity. J Biol Chem.

[35] Uwagawa T, Sakamoto T, Abe K, Okui N, Hata D, Shiba H, Futagawa Y, Aiba K, Yanaga K (2015). Phase I trial of S-1 every other day in combination with gemcitabine/cisplatin for inoperable biliary tract cancer. Cancer Chemother Pharmacol.

[36] Fujiwara Y, Shiba H, Iwase R, Haruki K, Furukawa K, Uwagawa T, Misawa T, Ohashi T, Yanaga K (2013). Inhibition of nuclear factor kappa-B enhances the antitumor effect of combination treatment with tumor necrosis factor-alpha gene therapy and gemcitabine for pancreatic cancer in mice. J Am Coll Surg.

[37] Fridman JS, Lowe SW (2003). Control of apoptosis by p53. Oncogene.

[38] Guo KY, Han L, Li X, Yang AV, Lu J, Guan S, Li H, Yu Y, Zhao Y, Yang J, Zhang H (2017). Novel proteasome inhibitor delanzomib sensitizes cervical cancer cells to doxorubicin-induced apoptosis via stabilizing tumor suppressor proteins in the p53 pathway. Oncotarget.

[39] Sachdeva UM, O’Brien JM (2012). Understanding pRb: toward the necessary development of targeted treatments for retinoblastoma. J Clin Invest.

[40] Gjidoda A, Henry RW (2013). RNA polymerase III repression by the retinoblastoma tumor suppressor protein. Biochim Biophys Acta.

[41] Van Cutsem E, Nordlinger B, Cervantes A, ESMO Guidelines Working Group (2010). Advanced colorectal cancer: ESMO Clinical Practice Guidelines for treatment. Ann Oncol.

[42] Zhang B, Wang D, Ji TF, Shi L, Yu JL (2017). Overexpression of lncRNA ANRIL up-regulates VEGF expression and promotes angiogenesis of diabetes mellitus combined with cerebral infarction by activating NF-kappaB signaling pathway in a rat model. Oncotarget.

[43] Leychenko A, Konorev E, Jijiwa M, Matter ML (2011). Stretch-induced hypertrophy activates NFkB-mediated VEGF secretion in adult cardiomyocytes. PLoS One.

[44] Pinto MP, Owen GI, Retamal I, Garrido M (2017). Angiogenesis inhibitors in early development for gastric cancer. Expert Opin Investig Drugs.

[45] San Miguel J, Weisel K, Moreau P, Lacy M, Song K, Delforge M, Karlin L, Goldschmidt H, Banos A, Oriol A, Alegre A, Chen C, Cavo M (2013). Pomalidomide plus low-dose dexamethasone versus high-dose dexamethasone alone for patients with relapsed and refractory multiple myeloma (MM-003): a randomised, open-label, phase 3 trial. Lancet Oncol.

[46] Shirai Y, Uwagawa T, Shiba H, Shimada Y, Horiuchi T, Saito N, Furukawa K, Ohashi T, Yanaga K (2017). Recombinant thrombomodulin suppresses tumor growth of pancreatic cancer by blocking thrombin-induced PAR1 and NF-kappaB activation. Surgery.

[47] Infante JR, Jones SF, Bendell JC, Spigel DR, Yardley DA, Weekes CD, Messersmith WA, Hainsworth JD, Burris HA (2011). A phase I, dose-escalation study of pomalidomide (CC-4047) in combination with gemcitabine in metastatic pancreas cancer. Eur J Cancer.

[48] Shimada Y, Kobayashi H, Kawagoe S, Aoki K, Kaneshiro E, Shimizu H, Eto Y, Ida H, Ohashi T (2011). Endoplasmic reticulum stress induces autophagy through activation of p38 MAPK in fibroblasts from Pompe disease patients carrying c.546G>T mutation. Mol Genet Metab.

[49] Nishioka N, Matsuoka T, Yashiro M, Hirakawa K, Olden K, Roberts JD (2011). Linoleic acid enhances angiogenesis through suppression of angiostatin induced by plasminogen activator inhibitor 1. Br J Cancer.

